# Intelligent Lung Support in the Intensive Care Unit (IntelliLung): study protocol for an international observational, prospective, multicentre study

**DOI:** 10.1136/bmjopen-2025-114851

**Published:** 2026-09-23

**Authors:** Raphael Theilen, Robert Huhle, Martin Scharffenberg, Franziska Fischer, Tim Kramer, Thea Koch, Lorenzo Ball, Lluis Blanch, Leonardo Sarlabous, Fernando Suárez-Sipmann, Konstanty Szuldrzynski, Milosz Jankowski, Julia Kalinka, Evelyn Trips, Barbara Djawid, Sarah Tsurkan, Valentina Janev, Miloš Nenadović, Dejan Paunović, Sahar Vahdati, Jaume M Castells, Andrea Kurz, Ahmed Hallawa, Ary Serpa Neto, Marcus J Schultz, Marcelo Gama de Abreu, Jakob Wittenstein, IntelliLung Consortium, Gomà G

**Affiliations:** 1 Department of Anesthesiology and Intensive Care Medicine, Pulmonary Engineering Group, Faculty of Medicine and University Hospital Carl Gustav Carus, TUD Dresden University of Technology, Dresden, Germany; 2 Anesthesia and Critical Care, San Martino Policlinico Hospital, Genoa, Italy; 3 Anesthesia and Intensive Care, Ospedale Policlinico San Martino, IRCCS per l’Oncologia e le Neuroscienze, Genova, Italy; 4 Critical Care Department, Parc Taulí Hospital Universitari, Institut d’Investigació I Innovació Parc Taulí (I3PT-CERCA), Sabadell, Spain; 5 Centro Investigación Biomédica en Red de Enfermedades Respiratorias (CIBERES), Instituto de Salud Carlos III, Madrid, Spain; 6 Institut d’Investigació i Innovació Parc Taulí (I3PT-CERCA), Instituto de Salud Carlos III, Sabadelle, Spain; 7 Intensive Care Medicine, Hospital Universitario de la Princesa. Universidad Autonoma de Madrid, Madrid, Spain; 8 Instituto de Investigación Sanitaria Hospital Universitario la Princesa, Universitario la Princesa, Madrid, Spain; 9 Department of Anaesthesiology and Intensive Care, National Medical Institute Ministry of Interior and Administration, Warsaw, Poland; 10 Coordination Centre for Clinical Trials, Faculty of Medicine Carl Gustav Carus, TUD Dresden University of Technology, Dresden, Germany; 11 Else Kroener Fresenius Center for Digital Health, TUD Dresden University of Technology, Dresden, Germany; 12 Institute Mihajlo Pupin, University of Belgrade, Belgrade, Serbia; 13 Nature-Inspired Machine Intelligence Group, Institute for Applied Informatics (InfAI), Dresden, Germany; 14 Better Care SL Head of Engineering & Company Co-Founder, Better Care, Sabadelle, Spain; 15 Rector, Medical University of Graz, Graz, Austria; 16 Department of Information Theory and Data Analytics, RWTH Aachen University, Aachen, Germany; 17 Australian and New Zealand Intensive Care Research Centre (ANZIC-RC), School of Public Health and Preventive Medicine, Monash University, Melbourne, Victoria, Australia; 18 Department of Intensive Care, Austin Hospital, Melbourne, Victoria, Australia; 19 Department of Critical Care, Melbourne Medical School, University of Melbourne, Austin Hospital, Melbourne, Victoria, Australia; 20 Department of Critical Care Medicine, Hospital Israelita Albert Einstein, Sao Paulo, Brazil; 21 Intensive Care, Amsterdam University Medical Centers, Amsterdam, Netherlands; 22 Mahidol Oxford Tropical Medicine Research Unit, Bangkok, Bangkok, Thailand; 23 Department of Intensive Care, University of Oxford, Oxford, UK; 24 Nuffield Department of Medicine, Mahidol University, Bangkok, Thailand; 25 Department of Anesthesia, General Intensive Care and Pain Management, Division of Cardiothoracic and Vascular Anesthesia & Critical Care Medicine, Medical University of Vienna, Vienna, Austria; 26 Division of Intensive Care and Resuscitation, Department of Anaesthesiology, Integrated Health Care Institute, Cleveland Clinic, Cleveland, Ohio, USA; 27 IntelliLung Consortium, Dresden, Germany

**Keywords:** Artificial Intelligence, Intensive Care Units, Adult intensive & critical care

## Abstract

**Introduction:**

Mechanical ventilation (MV) is lifesaving in the intensive care unit (ICU) but can cause complications if not individualised according to the patient’s needs. Artificial intelligence (AI)-driven decision support systems (AI-DSS) may theoretically optimise MV settings. This international observational, prospective, multicentre study aims to validate the IntelliLung AI-DSS in real clinical environments.

**Methods and analysis:**

In this study, patients aged ≥18 years requiring invasive MV for >24 hours are included. The primary objective is to evaluate the agreement between IntelliLung AI-DSS MV recommendations and the ventilator settings implemented by healthcare providers. The IntelliLung AI-DSS continuously analyses patient-specific data, including respiratory mechanics and gas exchange, to recommend optimal MV parameters. The primary endpoints are the relative time of matching ventilator settings for each (1) positive end-expiratory pressure, (2) fraction of inspired oxygen, (3) respiratory rate and (4) tidal volume during volume-controlled ventilation or inspiratory pressure (Pinsp) during pressure-controlled ventilation. Secondary endpoints include assessments of ventilator-free days and clinical decision-making practices. Patient-centred outcomes, such as quality of life and psychological stress, are also evaluated. Data collection spans ICU stay and follow-up at 30 and 180 days after enrolment. This trial is the first to validate the IntelliLung AI-DSS in a prospective, real-world clinical setting by comparing recommendations given by the IntelliLung AI-DSS to local standards of care. The results of the trial will serve as a foundation for future interventional studies to assess the IntelliLung AI-DSS impact on patient outcomes and ICU workflows. The study addresses a critical gap in the application of AI to intensive care, advancing personalised and evidence-based MV management.

**Ethics and dissemination:**

The TUD Medical Faculty Ethical Committee for clinical research approved the study on 4 November 2024 (File number Mono-EK-27907202). Additionally, the institutional review board at Sabadell, Madrid and Warsaw approved the study. IntelliLung is designed in accordance with the principles of the Declaration of Helsinki. The final main results will be published in a highly ranked, peer-reviewed scientific journal taking into account the recommendations of the International Committee of Medical Journal Editors.

**Trial registration number:**

NCT06595602.

**STRENGTHS AND LIMITATIONS OF THIS STUDY**

- This international, multicentre, prospective observational study design enhances the external validity and generalisability of findings across diverse intensive care unit populations and healthcare systems.
- The real-time validation of the artificial intelligence decision support system in routine clinical practice without influencing clinical decision-making ensures technical validity while maintaining adherence to local care standards.
- The broad inclusion of patient subgroups, including typically under-represented populations in intensive care research, strengthens the applicability and generalisability of the IntelliLung system recommendations across diverse patient demographics.
- The observational study design limits the ability to establish causal relationships between artificial intelligence recommendations and clinical outcomes, allowing only for assessment of associations and concordance with clinical practice.
- The study’s reliance on real-world clinical data collection across multiple international sites may introduce variability in data quality, documentation practices and potential selection bias despite standardised protocols.

## Introduction

 More than 40% of patients treated in an intensive care unit (ICU) require support for lung function, which is accomplished with mechanical ventilation (MV) and, in the most severe cases, extracorporeal gas exchange (ECLS, extracorporeal life support).^1^ Thereby, MV represents the principal life-saving therapy to support organ function in the ICU. However, inappropriate MV settings can aggravate or initiate lung injury, so-called ventilator-induced lung injury, impair haemodynamics and induce extra-pulmonary organ damage.^2 3^

Although clinical guidelines of protective MV represent an important aid in the attempt to mitigate ventilator-induced lung and distant organ injury,^4–6^ they provide only general rules and do not reproduce clinical experience that permits tailoring of MV to individual patient needs. Accordingly, non-individualised settings may result in an increased risk of lung injury in the majority of mechanically ventilated patients, since the broad knowledge and consideration of multiple information sources that are used by clinical experts cannot be easily reproduced. Competing treatment priorities can further hamper the urgent decision-making of attending clinical experts. Moreover, patients’ needs and clinical characteristics may change rapidly, requiring timely and appropriate titration of MV. Furthermore, recent studies showed that guidelines for protective MV are poorly followed worldwide.^7–9^ This has been ascribed to human factors, for example, lack of knowledge or expertise, time constraints to titrate MV in the face of personnel shortage and workload, and fatigue during night shifts.^10^

Manually optimising lung support is time-consuming and error-prone for ICU healthcare providers. Consequently, MV settings affect both the number of ventilation-free days and mortality.^11 12^ Artificial intelligence (AI) can be used to retrospectively learn from a patient’s condition in an attempt to improve the combination of settings and accomplish lung support with a reduced risk of ventilator-induced organ damage. An AI-based decision support system (AI-DSS) may offer advantages in circumstances where complex decisions are required to weigh potentially competing risks, depending on the physiological state of the patients.

We developed the IntelliLung AI-DSS to enhance the management of invasively ventilated ICU patients. Using reinforcement learning (RL)-AI-based algorithms, the system analyses patient-specific data and provides recommendations for setting key ventilator parameters, including positive end-expiratory pressure (PEEP), fraction of inspired oxygen (F_I_O₂), respiratory rate (RR) and tidal volume (V_T_) during volume-controlled ventilation modes, as well as pressure amplitude during pressure-controlled ventilation. Despite promising retrospective and simulation studies,^13–15^ few AI-DSS for MV have been prospectively validated in real-world, multicentre ICU settings.^16^

In view of these facts, we designed the international observational, prospective, multicentre Intelligent Lung Support in the Intensive Care Unit (IntelliLung) Trial, which aims to compare the degree of agreement of the recommendations delivered by the IntelliLung AI-DSS and the MV settings chosen by attending healthcare providers. The degree of agreement is calculated for PEEP, F_I_O_2_, RR and V_T_ or pressure amplitude.

## Methods and analysis

### Objectives and design

The IntelliLung trial is an international observational, prospective, multicentre study. In total, 530 patients will be included in the trial (see figure 1). The primary objective of this study is the prospective validation of the IntelliLung AI-DSS-Algorithm in its intended real-time critical care environment. The degree of agreement between the recommended MV settings by the IntelliLung AI-DSS and the actual ventilatory settings of the attending clinicians will be explored. However, this is an observational study, as the generated MV settings will not be disclosed to the clinicians and will not be applied during actual critical care. Thus, all participating patients will receive treatment according to the local standard of care. All study procedures will be conducted in accordance with ISO 14155, the current version of the Helsinki Declaration, and the European Medical Device Regulations (EU Regulation 745/2017). The Strengthening the Reporting of Observational Studies in Epidemiology (STROBE) Statement: guidelines for reporting observational studies^17^ will be followed.

**Figure 1:**
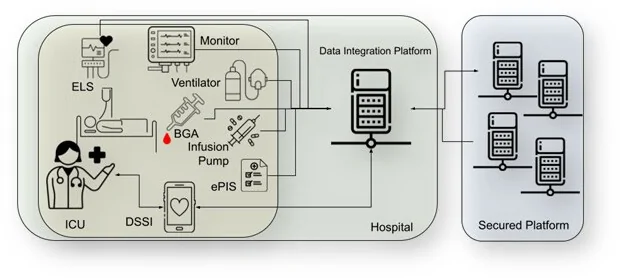
Artificial intelligence driven decision support systems interaction with ICU environment and data flow. BGA, blood gas analysis; DSSI, decision support system interface; ELS, extracorporeal life support; ePIS, electronic patient information system; ICU, intensive care unit.

## Medical device description

### Identification and description of the AI-DSS

The IntelliLung AI-DSS is a medical device software intended to monitor and recommend ventilator settings to qualified ICU healthcare professionals (HCP). It is designed for medical indications requiring invasive MV in the ICU, consistent with applicable international and European Union (EU) guidelines.^4 6 18^

As shown in figure 1, the device receives clinical data via the ICU’s data integration platform, including patient physical and demographic data, current vital signs, ventilation parameters, blood gas analysis, general laboratory results, fluid balance and medication. AI-based prediction models derive therapy suggestions from these data. The algorithm runs on a dedicated, on-site secured platform (SP), where recommendations are generated and stored. As the IntelliLung AI-DSS output is not displayed to treating clinicians during the study, no side effects or interactions are expected.

### Development of the software

The IntelliLung software is developed in accordance with IEC 62304:2016 (medical device software life-cycle processes), following the recommendations of MDCG 2019-11 and MDCG 2019-16 (cybersecurity guidance), in compliance with EU Regulation 2017/745 (Medical Device Regulation).

The recommendation-generating algorithm is a factored conservative Q-learning (CQL) model, trained on 22 April 2025 on a combined cohort derived from the MIMIC-IV,^19 20^ eICU^21 22^ and HiRID^23 24^ available on PhysioNet,^25^ using an 80% training 20% test split. The availability of parameters of the respective datasets is depicted in table 1. This approach reflects clinical reasoning at the bedside, where treatment decisions are necessarily based on the most recently available measurement until a new value is obtained. The training cohort and state-vector definition are summarised in tables 2 and 3. Model performance was assessed through retrospective internal validation including medical evaluation of action frequency distribution on the held-out test set, as shown in figure 2. While training data and performance log will be made publicly available at OpARA (https://opara.zih.tu-dresden.de), the full methodological details and validation results will be reported separately (see table 4).

**Table 1:** Data availability per patient and database

	Availability per patient (%)		
Parameter	HiRID	MIMIC-IV	eICU
Bodyweight	100	100	100
Height	100	100	100
Age	100	100	100
Serum chloride	97.5	97	94.5
Heart rate	100	100	100
White blood cell count	82.8	95	93.1
Arterial partial pressure of oxygen	96.7	88	75.7
Systolic blood pressure	100	75	100
Base excess	97.7	88	70.8
Arterial partial pressure of oxygen	96.7	88	75.1
Mean arterial blood pressure	100	99	99.8
Diastolic blood pressure	100	75	100
Serum potassium	97.5	98	95.4
International normalised ratio	73.7	90	53.1
pH value	96.7	94	73.9
Arterial oxygen saturation	97.7	54	96.5
Haemoglobin concentration	98.2	96	94.3
Serum sodium	97.5	97	94.8
Bicarbonate	97.7	96	95.2
Peripheral oxygen saturation	100	100	99.1
Fraction of inspired oxygen	99.9	100	89.4
Positive end-expiratory pressure	99.8	99	80.8
Respiratory rate	100	100	99.9
Tidal volume	99.9	99	78.9
Driving pressure	99.7	99	61.4

Relative availability of relevant parameters of all timepoints per observed patient.

**Table 2:** Training cohort IntelliLung algorithm

Characteristics	Training cohort	Testing cohort
No. of ICU stays	10 057	2515
Male	6712 (66.7%)	1725 (68.6%)
Age (years)	64 (54–77)	65 (54–77)
Body weight (kg)	82.0 (70.0–95.9)	82.0 (70.0–95.8)
Height (cm)	170.2 (166.0–178.0)	172.0 (166.0–178.0)
ICU mortality(%)	24.9	24.1
No. IMV events per patient	4140 (1500–9900)	4140 (1440–10 062)

Demographic data of the cohorts from the training and test datasets obtained from the public datasets MIMIC-IV, eICU and HiRID are shown as median (IQR).

ICU, Intensive care unit; IMV, invasive mechanical ventilation.

**Table 3:** State vector IntelliLung

Parameter groups	Parameter	Training cohort	Testing cohort
Action parameters	F_i_O_2_ (%)	44.5 (±14.2)	44.7 (±14.4)
	PEEP (cmH_2_O)	6.6 (±2.6)	6.6 (±2.6)
	RR (min^−1^)	20.1 (±6.4)	20.3 (±6.7)
	Vt (mL·kg^−1^)	7.7 (±1.8)	7.6 (±1.9)
	Driving pressure (cmH_2_O)	14.8 (±6.3)	14.7 (±6.5)
	Ventilation mode	VCV: 65.2% PCV: 34.8%	VCV: 64.1% PCV: 35.9%
Blood values	BE (mmol·L^−1^)	1.1 (±5.0)	0.9 (±5.2)
	Chloride (mmol·L^−1^)	107 (±6.6)	107.1 (±6.5)
	HaCO_3_ (mm Hg)	25.4 (±4.8)	25.3 (±4.8)
	Hb (mmol·L^−1^)	5.9 (±1)	5.9 (±1.1)
	INR	1.3 (±0.6)	1.4 (±0.6)
	PaCO_2_ (mm Hg)	38.9 (±8.5)	39.1 (±8.6)
	PaO_2_ (mm Hg)	101.2 (±44.9)	100.9 (±45.9)
	pH	7.4 (±0.1)	7.4 (±0.1)
	Potassium (mmol·L^−1^)	4.1 (±0.5)	4.1 (±0.5)
	SaO_2_ (%)	96.6 (±3.7)	96.5 (±3.5)
	Sodium (mmol·L^−1^)	140.1 (±5.6)	140.3 (±5.7)
	WBC (10^9^·L^−1^)	12.6 (±5.9)	12.5 (±5.9)
Vital parameters	Systolic BP (mm Hg)	117.1 (±24.1)	116.8 (±24.1)
	MAP (mm Hg)	76.6 (±15.4)	76.3 (±15.6)
	Diastolic BP (mm Hg)	58.8 (±13.5)	58.5 (±13.7)
	HR (min^−1^)	88.5 (±17.4)	89.3 (±17.9)
	SpO_2_ (%)	96.7 (±3.6)	96.6 (±3.7)

IntelliLung state vector parameters from the training and test datasets obtained from the public datasets MIMIC-IV, eICU and HiRID are shown as mean (±SD), if not indicated otherwise. Action parameters: ventilation parameters the IntelliLung AI-DSS is recommending settings for FIO_2_, PEEP, RR, HaCO_3_, Hba, PaCO_2_, PaO_2_, pHa, SaO_2_, MV, Pinsp, VT, MAP, HR, SpO_2_.

F_I_O_2_, inspiratory oxygen fraction; HaCO_3_, arterial bicarbonate; Hba, arterial haemoglobin; HR, heart rate; MAP, mean arterial blood pressure; MV, minute ventilation; PaCO_2_, arterial partial pressure of carbon dioxide; PaO2, arterial partial pressure of oxygen; PEEP, positive end-expiratory pressure; pHa, arterial pH; P_insp_, inspiratory airway pressure during pressure-controlled ventilation and plateau pressure during volume-controlled ventilation; RR, respiratory rate; SaO_2_, arterial oxygen saturation; SpO_2_, periphery oxygen saturation; V_T_, tidal volume.

**Figure 2:**
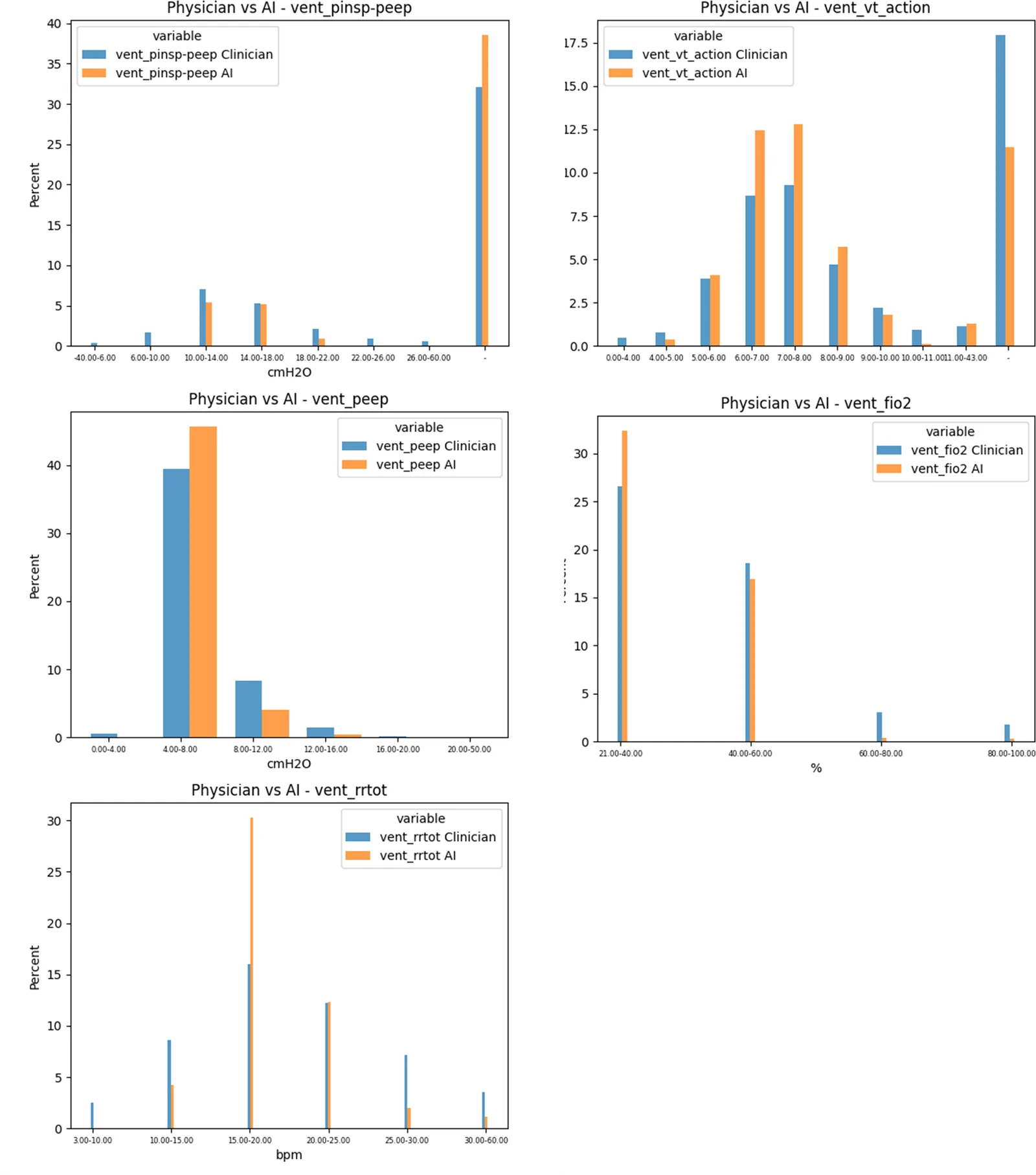
Relative frequency of the test dataset (physician) and policy (AI) action bins. AI, artificial intelligence; vent_fio2, inspiratory oxygen fraction (FIO₂); vent_peep, positive end-expiratory pressure (PEEP); vent_rrtot, total respiratory rate; vent_vtnorm, tidal volume normalised to ideal body weight (IBW); vent_vp, ventilation/driving pressure.

**Table 4:** Data sharing plan

Will individual deidentified participant data (IPD) be shared?	Yes
What data will be shared?	Deidentified individual participant-level data underlying the published results, together with the data dictionary/variable definitions. Direct identifiers are removed and a pseudonymisation key is held separately at the sponsor site. Only deidentified data leave the consortium
What related documents will be available?	Study protocol, statistical analysis plan and informed consent template
When will data become available, and for how long?	From publication of the study B results, for a minimum of 10 years (consistent with the project’s OpARA deposition commitment)
By what access criteria/mechanism?	IPD available on reasonable request to the corresponding author. Requesters submit a methodologically sound proposal and analysis plan. Access is granted under a data-sharing/use agreement, subject to GDPR and ethics-committee conditions, with re-use restricted to the approved purpose
Model data and source code	The training and validation datasets will be made openly available on OpARA, the Open Access Repository and Archive of the Saxon universities / TU Dresden (https://opara.zih.tu-dresden.de/), in accordance with the FAIR principles (https://www.go-fair.org/fair-principles/). These materials become available only after publication of the separate paper reporting model development and retrospective performance assessment. The repository record will be populated at that point. The source code of the deployed algorithm in study B is publicly available at (https://github.com/NIMI-research/INTELLILUNG_2)

GDPR, General Data Protection Regulation.

### Integration into the ICU environment

Clinical data are sent to a data integration platform (DIP; Better Care, Sabadell, Spain), which communicates with the SP (Mihajlo Pupin Institute, Serbia) that runs the AI-DSS algorithm. Both platforms are designed to operate independently of each hospital’s specific IT infrastructure, enabling deployment across diverse network and device environments. Although bedside devices, patient information systems and network architecture differ between participating centres, the DIP and SP provide a consistent, site-independent core.

### Study population

The investigators screen for patients aged ≥18 years undergoing invasive MV and foreseen to remain so for >24 hours. Patients expected to die within the next 48 hours, participating in an interventional MV trial, receiving MV with a closed-loop ventilation mode, dependence on the sponsor and/or investigator, and subjects who are currently imprisoned or otherwise in confinement ordered by law or other official authorities will be excluded from the trial.

### Study endpoints

The primary endpoint measures the proportion of time during which the ventilator settings chosen by the healthcare provider match those recommended by the IntelliLung AI-DSS. This proportion is calculated relative to the total time the IntelliLung AI-DSS is active for each patient, and is assessed for the following ventilatory settings: PEEP, F_I_O_2_, RR, ventilation mode (either volume-controlled ventilation/VCV or pressure-controlled ventilation/PCV) and either V_T_ during VCV or the pressure amplitude during PCV. Matching device settings are defined as current setting of the healthcare provider lying within the respective range recommended by the IntelliLung AI-DSS.

Secondary algorithm-related endpoints include:

- Direction of change (increase/decrease) in AI-DSS proposed MV settings as compared with direction of change in healthcare provider MV settings.
- Time between AI-proposed change of MV settings and change of MV settings by healthcare provider (time of extubation or liberation from invasive MV).
- Difference in parameters and magnitude of the deviation (∆DS) in diverging device settings of the IntelliLung AI-DSS and the healthcare provider (RR, F_I_O_2_, PEEP, V_T_ during VCV or inspiratory pressure amplitude during PCV).
- Current practices in clinical decision-making regarding MV by healthcare provider (RR, F_I_O_2_, PEEP, V_T_ during VCV/inspiratory pressure amplitude during PCV, time of extubation or liberation from invasive MV).

Secondary patient-related endpoints include:

- Weaning failure according to RESCUE-2-trial criteria^26^:
- Extubation with subsequent reintubation within 48 hours or subsequent increase of Continuous Positive Airway Pressure(CPAP)/PEEP and/or pressure support in patients with tracheal cannula. Successful liberation from ventilation according to RESCUE-2-trial criteria^26^ is defined as follows:
  - Patients with endotracheal tube: spontaneous breathing trial (SBT) with CPAP/PEEP 0, pressure support/P_insp_ 0 cmH_2_O for 30–120 min or extubation and no subsequent reintubation within 48 hours.
  - Patients with tracheal cannula SBT with CPAP/PEEP 0 cmH_2_O, pressure support/P_insp_ 0 cmH_2_O or disconnection from the ventilator for 24 hours and no subsequent increase in CPAP/PEEP and/or pressure support.
- Ventilator-free days at day 30 after initiation of IntelliLung AI-DSS, defined as the number of calendar days that a patient was alive and free from invasive ventilation. A calendar day is defined as ‘free from invasive ventilation’ if no invasive ventilation was applied or invasive ventilation lasted less than 6 hours.
- In-hospital mortality, mortality at day 30, mortality at day 180 after initiation of the IntelliLung AI-DSS.
- Questionnaires (incl. quality of life):
  - 5-Level Quality of Life score (‘EQ-5D-5L’).^27^
  - Hospital Anxiety and Depression Scale (‘HADS’).^28^
  - Multi-Independence Dimension questionnaire (‘MIND’).^29^
- Clinical assessment scores:
  - Glasgow Coma Scale (GCS).^30 31^
  - Sequential Organ Failure Assessment (SOFA) score.^32^
  - Acute Physiology and Chronic Health Evaluation (APACHE II) score.^33^
  - Richmond Agitation-Sedation-Scale (RASS).^34^
    Exploratory endpoints include:

- Ventilatory ratio.^35 36^
- Incidence and time of ‘missed opportunities’, defined as situations in which non-lung-protective settings are defined as VT of >8 mL·kg^-1^ predicted body weight and PEEP of <5 cmH_2_O (both must be met).
- Current practices in clinical decision-making regarding ECLS setting by healthcare provider.

### Study visits and data collection

Patients are screened according to inclusion and exclusion criteria. All eligible patients are registered in a screening log file by each site. As patients undergoing MV are usually unable to provide informed consent, it is obtained either via a legally authorised representative or a consultant physician, according to the respective national regulations. On the day of enrolment, demographic and baseline variables are collected including demographic data (eg, age and ethnic background), medical history and current medical status such as use of vasopressors, functional status, smoking status, aspiration, pneumothorax, high-risk surgery or high-risk trauma, alcohol abuse, current chemotherapy, active cancer, chronic kidney failure including stage and renal replacement therapy status, liver cirrhosis including Child-class, intracranial injury or tumour, immunosuppression status, pulmonary status (ARDS according to Berlin definition,^37^ chronic obstructive pulmonary disease, including steroids and/or inhalation therapy use, asthma including steroids and/or inhalation therapy use), cardiovascular status (heart failure according to the New York Heart Association, coronary heart disease according to Canadian Cardiovascular Society, atrial flutter/fibrillation, arterial hypertension), and metabolic status (diabetes mellitus). After study inclusion, patients are visited daily during their stay in the study site’s ICU until liberation from invasive MV or discharge from the trial site ICU. A further visit is performed within 72 hours after extubation. Recorded data includes vital signs (eg, pulse frequency), arterial blood pressure, temperature, peripheral oxygen saturation, respiratory signals including ventilator setting (PEEP, mean airway pressure, pressure amplitude during PCV, V_T_, RR, F_I_O_2_), laboratory biochemical results (blood concentration of calcium, chloride, magnesium, potassium, sodium, blood urea nitrogen, creatinine, albumin, serum glutamic oxaloacetic transaminase, serum glutamic-pyruvic transaminase, total bilirubin, lactate, glucose), blood gas analysis (base excess, bicarbonate, pH value) and haematological report (small blood count, including haemoglobin and haematocrit). Extubation/liberation from the ventilator is defined as removal of the endotracheal tube or, in patients with tracheostomy, a pressure support of 0 cmH_2_O for at least 48 hours (figure 3). An overview of the visit schedule is given in table 5.

**Figure 3:**
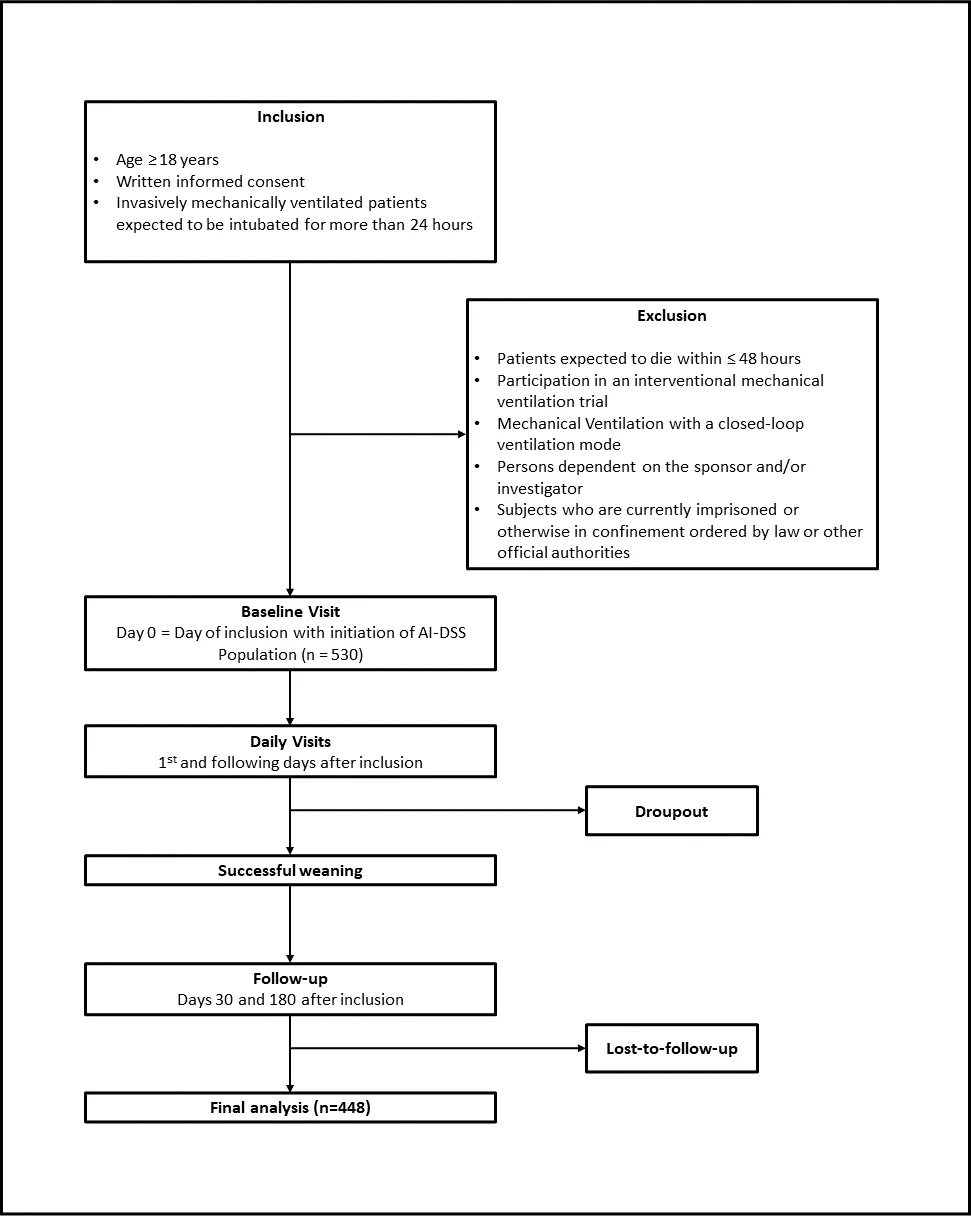
Consolidated Standards of Reporting Trials (CONSORT) diagram for the IntelliLung trial. AI-DDSS, artificial intelligence driven decision support systems; Exclusion, exclusion criteria; Inclusion, inclusion criteria.

**Table 5:** Visit schedule

	Visit 1: baseline visit, day 0	Visit 2: day 1−x: during mechanical ventilation (MV)	Visit 3 (follow-up): within 72 hours after liberation from MV- before discharge	Visit 4 (follow-up): day 30 (+5)	Visit 5 (follow-up): day 180 (+14)
Inclusion/exclusion criteria	X				
Informed consent (LAR or consultant physician*; patient**)	X*		X**		
Demographics	X				
Relevant medical history (incl. concomitant diseases)	X				
Reason for interruption of data flow	X	X			
SOFA (incl. GCS)	X	X	X		
APACHE-II	X	X	X		
RASS	X	X	X		
Vital signs	X	X	X		
Respiratory signals	X	X	X		
Ventilator settings	X	X	X		
ELS settings/parameters	X	X	X		
Adjunctive therapies***	X	X	X		
Laboratory parameter	X	X	X		
Point-of-care (blood gas analysis)	X	X	X		
Mortality	X	X	X	X	X
Duration of MV	X	X	X		
Re-initiation of invasive MV			X	X	X
EQ-5D-5L			X	X	X
HADS			X	X	X
MIND			X		

Parameters obtained at specific visit: LAR, SOFA, GCS, APACHE II, RASS, ECLS, EQ-5D-5L, HADS, MIND.

APACHE II, Acute Physiology And Chronic Health Evaluation Score II; ECLS, Extracorporeal life support; EQ-5D-5L, 5-Level Quality of Life score; GCS, Glasgow Coma Scale (obtained at least every x72 hours); HADS, Hospital Anxiety and Depression Scale; LAR, legally authorised representative; MIND, Multi-Independence Dimension questionnaire; RASS, Richmond Agitation Sedation Scale; SOFA, Sepsis-related Organ Failure Assessment score.

If patient consent is given, within 72 hours after successful liberation from the ventilator, instances of re-initiation of invasive MV, as well as patients’ ICU experience, shall be obtained via different questionnaires, including the EQ-5D-5L, the HADS and the MIND. On day 30 and 180 after study enrolment, survival status will be recorded, and patients will be sent the HADS and EQ-5D-5L questionnaires via mail. The number of ventilator-free days at day 30 will be recorded. Day 180 is the last day of follow-up; accordingly, patients still admitted to the hospital will be last visited on that day. Discharged patients will be contacted by phone.

### Study dropouts

Participation in the trial is voluntary. Patients or legally authorised representatives have the right to withdraw consent to the study at any time, for any reason, without any consequence for further medical treatment. If possible, the reasons and circumstances for study discontinuation will be documented in the electronic case report form (CRF).

### Handling of data

Monitoring in this investigation will be performed in a risk-based manner, including site initiation and interim monitoring. During the study period, centralised monitoring processes combined with on-site visits will be performed to ensure adherence to the investigational plan, and provide high data quality. The monitor must treat all information confidentially and respect and observe the participant’s right to privacy. Patient data are collected in pseudonymous form using a patient (identification) number. Study data obtained manually by local investigators are stored in the eCRF using MACRO^38^ hosted at the Clinical Trial Coordination Centre (KKS) of the TUD Dresden University of Technology, Germany.

Following pseudonymisation and parameter selection, the recorded health data from the study sites patient data management system and ICU devices of study participants are collected using the BC Link product supplied by Better Care as DIP. The IntelliLung AI-DSS algorithm itself is run on the SP provided by the Mihajlo Pupin Institute, Serbia. Study-relevant data will be recorded in the DIP and subsequently sent to the SP running the AI-DSS to derive and store the MV recommendations for analysis. Both the DIP and SP platforms operate in isolation from the production hospital network. For auditing, bug fixing and service of both the DIP and the SP, representatives of the respective developers have personalised Virtual Private Network access granted exclusively (ensured by dedicated firewall rules). A computer will be connected at the end of the study to the SP for data extraction for offline post-processing and parameter calculation. Healthcare providers will not be aware of the recommendations. All data will be stored at the SP side. Data will be recorded until 72 hours after successful weaning or discharge (whichever occurs first). In this regard, successful weaning will be defined as extubation in the case of intubated patients or liberation from active respiratory support in the case of tracheotomised patients. Any re-initiation of invasive MV will be documented during the ICU stay.

### Sample size calculations

The distribution and in particular the median of the degree of agreement of the four primary endpoints will be estimated along with CIs. A global confidence level of 95% and an accuracy of ±1/8 of the SD for normally distributed data are aimed at. Following the Bonferroni method,^39^ the individual confidence level for each of the four primary endpoints is 98.75%. With a sample size of 448 patients, a 95% probability of obtaining the desired precision is reached when estimating the mean for normally distributed data. Assuming a drop-out rate of about 15%, 530 patients need to be recruited in total. Sample size was calculated by SAS software V.9.4 (SAS Institute, Cary, USA, procedure POWER).

According to unpublished pilot data, 1/8 of SD corresponds approximately to 4% for agreement of F_I_O_2_, 3% for agreement of PEEP and 2% for agreement of RR.

In addition, data from a previous retrospective study showed that agreement endpoints are not normally distributed. Given a sample size of 448 and a significance level of 1.25%, the lower and upper limits of the distribution-free CI for the median equal the 198th and 251st ranks, that is, 44th and 56th percentiles. Simulations were performed to assess the precision that can be achieved with the given number of cases for estimation of distribution-free CIs for medians^40^ from skewed data. These simulations result in an expected precision of ±6.8% for agreement of F_I_O_2_, ±2.4% for agreement of PEEP and ±2.9% for agreement of RR.

### Statistical analysis

#### Primary endpoint

In this observational trial, no statistical hypotheses will be tested. The primary endpoint is therefore estimative and exploratory in nature. No a priori threshold of agreement is pre-specified as denoting successful performance, as the analysis aims to characterise the distribution of agreement rather than to test it against a hypothesised value. As the primary objective of this study is the prospective validation of the IntelliLung AI-DSS, the agreement of MV settings of the attending healthcare provider and recommendations by AI-DSS will be investigated by estimating the distribution of the four parameters included in the primary endpoint (relative time of same device settings of PEEP, F_I_O_2_, RR, V_T_ during VCV and P_insp_ during PCV) in the full analysis set. The full analysis set comprises all included patients with informed consent undergoing invasive MV. First, the distribution of all four primary parameters is described (boxplot; median, lower and upper quartile, minimum and maximum; mean and SD). A two-sided global confidence level is established at 95%. Following the Bonferroni method,^39^ the individual confidence level is 98.75% for each of the four primary parameters. According to unpublished pilot data, we do not expect a normal distribution for any primary parameter. Thus, a 98.75% two-sided distribution-free CI for the median will be reported. A certain amount of degree is expected and will be investigated in an explorative manner (secondary endpoint ‘Difference in parameters and magnitude of the deviation’).

#### Secondary endpoints

Secondary endpoints are analysed in a descriptive and explorative way. No further adjustment of the significance level is planned. Algorithm-related secondary endpoints are reported as a description of the distribution of all these secondary endpoints. The endpoint ‘Current practices in clinical decision making’ will be analysed by a description of the mentioned MV parameters chosen by local attending HCPs. Patient-related secondary endpoints are reported as absolute and relative frequencies (need for re-initiation of invasive MV (weaning failure)) and as a description of distribution (ventilator-free days until day 30, questionnaire MIND). In-hospital mortality, mortality at day 30, and mortality at day 180 will be assessed using the Kaplan-Meier method (in case of lost-to-follow-up cases) or absolute and relative frequencies in case of good adherence to protocol regarding follow-up observation. The quality-of-life assessment via questionnaires (EQ-5D-5L, HADS) and clinical assessment scores (GCS, SOFA score, APACHE II Score, RASS) are analysed by mixed models with patients as a random factor.

For exploratory endpoints, the distributions are described. The course of ventilator ratio is reported by a mixed model with the patient as a random factor. The incidence and time of ‘missed opportunities’ are described by reporting the number of events, and the absolute and relative frequency of patients concerned.

#### Adjusted and subgroup analyses

The influence of the amount of agreement between AI-DSS and HCP will be investigated for the secondary endpoints: mortality (in-hospital, day 30 and day 180) and VFD until day 30 in an explorative and hypothesis-generating manner. The influence will be investigated by regression models and endpoints will be described and compared between groups, with a high agreement between AI-DSS and healthcare providers and those with low agreement.

### Trial organisation

Dresden University of Technology is the sponsor of the trial. The Department of Anesthesiology and Intensive Care Medicine at the Faculty of Medicine and University Hospital Carl Gustav Carus is the coordinating centre. The trial is managed by a team consisting of the principal coordinating investigator of the IntelliLung-project (JW), the project managers (RT, FF), the statisticians (RH, ET), the study management as well as data management (KKS), clinical monitors and the Senior Scientific Advisor (MGdA). Monitoring tasks include the verification that the site meets the requirements for the conduct of the clinical investigation (subject population, equipment, etc), verification of informed consent forms, random source data verification as well as checking the Investigator Site File for completeness and currency. JW acts as guarantor for this study.

The Critical Care Department at Parc Taulí Hospital Universitari (Spain), the Department of Intensive Care Medicine at the Hospital Universitario de La Princesa (Spain) and Department of Anaesthesiology and Intensive Care at National Medical Institute of the Ministry of Interior and Administration (Poland) are collaborating institutions, each with nominated principal investigators, who are academic leads.

This trial will comply with the current, approved version of the protocol. Any significant changes that could affect the study or its participants will be reviewed and subsequently approved by the ethics committee at Dresden University of Technology. Amendments will be approved prior to implementation. Approval and updated protocols will be disseminated to all relevant institutions promptly.

### Patient and public involvement and dissemination plans

It was not possible to involve patients or the public in the design, conduct and reporting of the study. After the end of the clinical investigation, the statistical evaluation is performed first and then the final report is prepared, which contains all essential results of the investigation and meets the requirements of Annex D ISO 14155. This report is submitted to the responsible higher federal authority and the ethics committee within 1 year after the end of the clinical trial. The final main results will be published in a highly ranked, peer-reviewed scientific journal taking into account the recommendations of the International Committee of Medical Journal Editors (ICMJE). Preference shall generally be given to publication of the entire clinical investigation. Only the writing committee, which will be constituted during the course of the study may prepare results for the main publication. Any other arrangements must be agreed with the principal coordinating investigator. Investigators of this study are not allowed to carry out their own uncoordinated sub-analyses or partial analyses or to publish results in any way prior to the main publication.

### Ethics and dissemination

The study was approved by the Ethics Committee of the Medical Faculty of Technische Universität Dresden (File No. Mono-EK-27907202, 4 November 2024) as the coordinating ethics committee. Site-specific approvals were obtained from: The Komisja Bioetyczna Państwowego Instytutu Medycznego MSWiA, Warsaw, Poland (Decision No. 81/2024, 19 November 2024); the Comité de Ética de la Investigación con Medicamentos, Hospital Universitario de La Princesa, Madrid, Spain (Registry No. 5851, 30 January 2025); and the Comité de Ética de Investigación Clínica, Parc Taulí I3PT, Sabadell, Spain (Approval No. 2025/6030, 7 July 2025). The study is conducted in accordance with the Declaration of Helsinki and ICH-GCP guidelines and is registered on ClinicalTrials.gov (NCT06595602).^41 42^ The final primary results will be published in an appropriately ranked, peer-reviewed scientific journal in accordance with the recommendations of the International Committee of Medical Journal Editors (ICMJE). Authorship will follow ICMJE criteria. Pseudonymised participant-level data and analysis code will be made available on reasonable request to the corresponding author following publication, subject to applicable data protection regulations (including General Data Protection Regulation). Secondary analyses and subgroup findings will be reported in subsequent publications.

## Discussion

The IntelliLung trial was designed to prospectively validate the IntelliLung AI-DSS in real-time clinical settings to ensure its recommendations align with current clinical standards of care and are reproducible in a clinical setting. In this context, validation refers specifically to technical and operational validation: confirming that an algorithm trained and tested on retrospective data can generate stable, plausible and guideline-consistent recommendations when deployed against prospectively acquired, real-time clinical data. However, it does not denote validation of clinical efficacy or of an effect on patient outcomes, which is explicitly beyond the scope of this observational study and the subject of future interventional trials. We therefore use the agreement between AI-DSS recommendations and clinician-selected ventilator settings not as a measure of correctness against an objective ground-truth, but as a reproducible, clearly defined reference reflecting routine expert decision-making across four leading European ICUs as the current standard of MV management.

Unlike its prior development phase, where the IntelliLung AI-DSS was trained on retrospective data, its use in a clinical environment presents unique challenges. These include handling real-time data without manual pre-processing: thus, the data might deviate from the training data for various reasons, including a low degree of standardisation, or underrepresented patient sub-cohorts compared with the retrospective training data, which in turn may unpredictably influence the system’s recommendations.^43 44^ However, these challenges are not unique to the IntelliLung AI-DSS, as an increasing number of machine learning models have shown promising performance working with retrospective data,^45^ yet validation in clinical settings remains rare.^46^ Thus, before the effect of AI-DSS MV recommendations on patient outcomes can be assessed, its performance during training and retrospective testing must be reproducible in real-time clinical environments. Therefore, this trial is non-interventional, that is, observational, in design.

During the study, IntelliLung AI-DSS recommendations are not shared with healthcare providers, ensuring that patient care remains entirely in accordance with local standards and that there is no additional medical risk to participants compared with standard practice. All AI-generated recommendations are securely stored within the study’s IT infrastructure and are not accessible to clinical staff. This approach prevents any influence of the AI-DSS on clinical decision-making and guarantees that all therapy follows established local protocols. Because participation does not introduce extra risk, the eligibility criteria are intentionally broad, allowing the inclusion of typically underrepresented subgroups, such as pregnant and breastfeeding patients, who may have distinct therapeutic requirements. This inclusive strategy enables the evaluation of IntelliLung AI-DSS across a diverse ICU population, thereby strengthening the generalisability and subgroup-specific applicability of its recommendations.

Although the present study does not assess the clinical implementation of IntelliLung AI-DSS recommendations, the quality of these recommendations is systematically evaluated by comparing them to both local standards of care and the boundaries established by current international guidelines.^4 6 18^ While these guidelines represent the best available evidence, they frequently lack specific thresholds, leaving many ventilatory decisions to the discretion and expertise of the attending clinician. As a result, guideline-based assessment alone is insufficient for comprehensive validation of the AI-DSS. To address this, the study also benchmarks the IntelliLung AI-DSS derived recommendations against actual clinical practice across four leading European ICUs. This dual approach enables a more nuanced evaluation of the AI-DSS, capturing both its alignment with evidence-based recommendations and its adaptability to real-world clinical decision-making. It is important to note that local standards of care may diverge from established international guidelines.^7^ Crucially, neither current clinical practice nor existing guidelines constitute a true gold standard for MV with clearly defined safe thresholds for all relevant ventilation parameters. International guidelines, including lung-protective and ARDSNet-derived strategies, frequently lack these precisely defined thresholds and leave considerable discretionary leeway to clinicians during MV management.^4 6 19^ Clinician-selected settings, by contrast, represent a concrete, unambiguous and clinically meaningful treatment strategy actually delivered to patients, making them a pragmatic and interpretable reference against which the behaviour of the AI-DSS can be characterised. Consequently, discrepancies between IntelliLung AI-DSS recommendations and local clinical practice should not be interpreted as evidence of inferior system performance. In some instances, such differences may indicate that the AI-DSS is promoting safer or more evidence-based practices than those routinely implemented. Therefore, complete concordance between the AI-DSS and local standard of care is neither anticipated nor necessarily desirable. Instead, systematic analysis of these deviations can yield valuable insights into the potential added value of the IntelliLung AI-DSS. For example, situations where the AI-DSS aligns with guideline-based recommendations while clinical practice does not may highlight opportunities for improving patient management. Additionally, exploring the relationship between such discrepancies and patient outcomes, including duration of ventilation or clinical endpoints, will help to elucidate the clinical relevance and potential impact of the IntelliLung AI-DSS in real-world settings.

The ventilator parameters selected for recommendation by the IntelliLung AI-DSS were chosen to encompass the most clinically significant and modifiable aspects of MV, while ensuring compatibility with both retrospective data used for algorithm training and the majority of commonly employed ventilation modes. V_T_ was included due to its well-established association with ventilator-induced lung injury (VILI) and its central role in determining minute ventilation.^47^ In pressure-controlled ventilation modes, inspiratory pressure was incorporated as it primarily dictates the resulting V_T_. Respiratory rate was selected because it, together with V_T_, governs minute ventilation and thus directly influences carbon dioxide elimination. The recommended parameters include F_I_O₂ and PEEP to optimise oxygenation and minimise the risk of further lung injury. This selection reflects current evidence and guideline priorities for lung-protective ventilation strategies. In addition, the study also incorporates patient-reported outcomes to capture the broader impact of invasive MV. Patient experiences are systematically assessed using validated quality of life and psychological well-being questionnaires at multiple time points, shortly after, as well as one and 6 months following liberation from MV.^23–25 27–29 44–46 48–50^ This approach ensures that the evaluation of the IntelliLung AI-DSS extends beyond technical performance to include patient-centred outcomes, providing a comprehensive assessment of its potential benefits in clinical practice.

In summary, the IntelliLung international, prospective, observational, multicentre trial is the first study to validate an AI-DSS for MV setting recommendations in real-time in a clinical environment. The study will demonstrate whether the IntelliLung algorithm, which was trained on retrospective data, can produce recommendations and to which degree these align with the current standard of care MV practice. The findings of this trial shall pave the way for future testing of the effect of the IntelliLung AI-DSS on patient outcomes.

### Trial status

The current protocol is Version 4.0F, dated 1 September 2025. The IntelliLung trial is currently recruiting patients. Recruitment started in May 2025. The estimated completion date is the end of 2027.

## References

[1] Abrams D, Schmidt M, Pham T (2020). Mechanical Ventilation for Acute Respiratory Distress Syndrome during Extracorporeal Life Support. Research and Practice. Am J Respir Crit Care Med.

[2] Cortes-Puentes GA, Oeckler RA, Marini JJ (2018). Physiology-guided management of hemodynamics in acute respiratory distress syndrome. Ann Transl Med.

[3] Spieth PM, Bluth T, Gama De Abreu M (2014). Mechanotransduction in the lungs. Minerva Anestesiol.

[4] Fichtner F, Mörer O, Laudi S (2017). S3-Leitlinie „Invasive Beatmung und Einsatz extrakorporaler Verfahren bei akuter respiratorischer Insuffizienz. DIVI.

[5] Rochwerg B, Brochard L, Elliott MW (2017). Official ERS/ATS clinical practice guidelines: noninvasive ventilation for acute respiratory failure. Eur Respir J.

[6] Grasselli G, Calfee CS, Camporota L (2023). ESICM guidelines on acute respiratory distress syndrome: definition, phenotyping and respiratory support strategies. Intensive Care Med.

[7] Bellani G, Laffey JG, Pham T (2016). Epidemiology, Patterns of Care, and Mortality for Patients With Acute Respiratory Distress Syndrome in Intensive Care Units in 50 Countries. JAMA.

[8] Pisani L, Algera AG, Serpa Neto A (2021). Epidemiological Characteristics, Ventilator Management, and Clinical Outcome in Patients Receiving Invasive Ventilation in Intensive Care Units from 10 Asian Middle-Income Countries (PRoVENT-iMiC): An International, Multicenter, Prospective Study. Am J Trop Med Hyg.

[9] Pisani L, Algera AG, Neto AS (2022). Geoeconomic variations in epidemiology, ventilation management, and outcomes in invasively ventilated intensive care unit patients without acute respiratory distress syndrome: a pooled analysis of four observational studies. Lancet Glob Health.

[10] Cortegiani A, Gregoretti C, Neto AS (2019). Association between night-time surgery and occurrence of intraoperative adverse events and postoperative pulmonary complications. Br J Anaesth.

[11] Cavalcanti AB, Suzumura EA, Laranjeira LN (2017). Effect of Lung Recruitment and Titrated Positive End-Expiratory Pressure (PEEP) vs Low PEEP on Mortality in Patients With Acute Respiratory Distress Syndrome: A Randomized Clinical Trial. JAMA.

[12] Constantin J-M, Jabaudon M, Lefrant J-Y (2019). Personalised mechanical ventilation tailored to lung morphology versus low positive end-expiratory pressure for patients with acute respiratory distress syndrome in France (the LIVE study): a multicentre, single-blind, randomised controlled trial. Lancet Respir Med.

[13] Peine A, Hallawa A, Bickenbach J (2021). Development and validation of a reinforcement learning algorithm to dynamically optimize mechanical ventilation in critical care. NPJ Digit Med.

[14] Gupta P, Pearce AK, Pham T (2025). Artificial intelligence-driven decision support for patients with acute respiratory failure: a scoping review. Intensive Care Med Exp.

[15] Otten M, Jagesar AR, Dam TA (2024). Does Reinforcement Learning Improve Outcomes for Critically Ill Patients? A Systematic Review and Level-of-Readiness Assessment. Crit Care Med.

[16] Patel BV, Mumby S, Johnson N (2024). A randomized control trial evaluating the advice of a physiological-model/digital twin-based decision support system on mechanical ventilation in patients with acute respiratory distress syndrome. Front Med (Lausanne).

[17] Equator Network STROBE: strengthening the reporting of observational studies in epidemiology. https://www.equator-network.org/reporting-guidelines/strobe/.

[18] Fan E, Del Sorbo L, Goligher EC (2017). An Official American Thoracic Society/European Society of Intensive Care Medicine/Society of Critical Care Medicine Clinical Practice Guideline: Mechanical Ventilation in Adult Patients with Acute Respiratory Distress Syndrome. Am J Respir Crit Care Med.

[19] Johnson A, Bulgarelli L, Pollard T (2026). MIMIC-IV. PhysioNet.

[20] Johnson AEW, Bulgarelli L, Shen L (2023). MIMIC-IV, a freely accessible electronic health record dataset. Sci Data.

[21] Pollard TJ, Johnson AEW, Raffa J (2017). The eICU Collaborative Research Database. PhysionetOrg.

[22] Pollard TJ, Johnson AEW, Raffa JD (2018). The eICU Collaborative Research Database, a freely available multi-center database for critical care research. Sci Data.

[23] Faltys M, Zimmermann M, Lyu X (2026). HiRID, a high time-resolution ICU dataset. PhysioNet.

[24] Hyland SL, Faltys M, Hüser M (2020). Early prediction of circulatory failure in the intensive care unit using machine learning. Nat Med.

[25] Pollard T, Moody BE, Lehman LH (2026). PhysioNet as a global platform for biomedical research. Nat Health.

[26] Dres M, de Abreu MG, Merdji H (2022). Randomized Clinical Study of Temporary Transvenous Phrenic Nerve Stimulation in Difficult-to-Wean Patients. Am J Respir Crit Care Med.

[27] Herdman M, Gudex C, Lloyd A (2011). Development and preliminary testing of the new five-level version of EQ-5D (EQ-5D-5L). Qual Life Res.

[28] Zigmond AS, Snaith RP (1983). The hospital anxiety and depression scale. Acta Psychiatr Scand.

[29] Winck JC, Gilet H, Kalin P (2019). Validation of the Multi-INdependence Dimensions (MIND) questionnaire for prolonged mechanically ventilated subjects. BMC Pulm Med.

[30] Mehta R (2019). GP trainee1, Chinthapalli K, consultant neurologist2. Glasgow coma scale explained. BMJ.

[31] Teasdale G, Jennett B (1974). ASSESSMENT OF COMA AND IMPAIRED CONSCIOUSNESS. Lancet.

[32] Vincent JL, Moreno R, Takala J (1996). The SOFA (Sepsis-related Organ Failure Assessment) score to describe organ dysfunction/failure: On behalf of the Working Group on Sepsis-Related Problems of the European Society of Intensive Care Medicine (see contributors to the project in the appendix). Intensive Care Med.

[33] Knaus WA, Draper EA, Wagner DP (1985). APACHE II: a severity of disease classification system. Crit Care Med.

[34] Pop MK, Dervay KR, Dansby M (2018). Evaluation of Richmond Agitation Sedation Scale (RASS) in Mechanically Ventilated in the Emergency Department. Adv Emerg Nurs J.

[35] Papoutsi E, Giannakoulis VG, Routsi C (2023). Association between ventilatory ratio and mortality persists in patients with ARDS requiring prolonged mechanical ventilation. Intensive Care Med.

[36] Sinha P, Calfee CS, Beitler JR (2019). Physiologic Analysis and Clinical Performance of the Ventilatory Ratio in Acute Respiratory Distress Syndrome. Am J Respir Crit Care Med.

[37] Ranieri VM, Rubenfeld GD, Thompson BT (2012). Acute respiratory distress syndrome: the Berlin Definition. JAMA.

[38] Ennov https://en.ennov.com/solutions/clinical/macro/.

[39] Holm S (1979). A Simple Sequentially Rejective Multiple Test Procedure. Scand J Stat.

[40] Hahn GJ, Meeker WQ (1991). Statistical Intervals: A Guide for Practitioners.

[41] (2013). World Medical Association Declaration of Helsinki: ethical principles for medical research involving human subjects. JAMA.

[42] Bharati V, Rajaram S, Prakash A (2025). International Council for Harmonisation E6 (R3): The Good Clinical Practice, recent developments, and global perspective. Indian J Pharmacol.

[43] Cohen JP, Cao T, Viviano JD (2021). Problems in the deployment of machine-learned models in health care. CMAJ.

[44] Lovejoy CA, Buch V, Maruthappu M (2019). Artificial intelligence in the intensive care unit. Crit Care.

[45] Baur D, Gehlen T, Scherer J (2022). Decision support by machine learning systems for acute management of severely injured patients: A systematic review. Front Surg.

[46] Sande D, Genderen ME, Huiskens J (2021). Moving from bytes to bedside: a systematic review on the use of artificial intelligence in the intensive care unit. Intensive Care Med.

[47] (2000). Ventilation with Lower Tidal Volumes as Compared with Traditional Tidal Volumes for Acute Lung Injury and the Acute Respiratory Distress Syndrome. N Engl J Med.

[48] Weiss DS, Wilson JP, Tang CS (2007). Cross-Cultural Assessment of Psychological Trauma and PTSD.

[49] Cornock MA (1998). Stress and the intensive care patient: perceptions of patients and nurses. J Adv Nurs.

[50] Rosner R, Hagl M (2008). Die revidierte Impact of Event-Skala (IES-R). Psychosom Konsiliarpsychiatr.

