# Supplementary material for: Intelligent Lung Support in the Intensive Care Unit (IntelliLung): study protocol for an international observational, prospective, multicentre study
Source: BMJ Open. 2026 Sep 23;16(9):e114851. doi: 10.1136/bmjopen-2025-114851 (PMC13630060; doi:10.1136/bmjopen-2025-114851)
Supplement: Supplementary file 1 [file bmjopen-2025-114851.reviewer_comments.pdf]

PEER REVIEW HISTORY

BMJ Open publishes all reviews undertaken for accepted manuscripts. Reviewers are asked to complete a checklist review form and are provided with free text boxes to elaborate on their assessment. These free text comments are reproduced below.

ARTICLE DETAILS

Title (Provisional)

Intelligent Lung Support in the Intensive Care Unit (IntelliLung): Study Protocol for an International Observational, Prospective, Multicentre Study

Authors

Theilen, Raphael; Huhle, Robert; Scharffenberg, Martin; Fischer, Franziska; Kramer, Tim; Koch, Thea; Ball, Lorenzo; Blanch, Lluis; Sarlabous, Leonardo; Suárez-Sipmann, Fernando; Szuldrzynski, Konstanty; Jankowski, Milosz; Kalinka, Julia; Trips, Evelyn; Djawid, Barbara; Tsurkan, Sarah; Janev, Valentina; Nenadović, Miloš; Paunović, Dejan; Vahdati, Sahar; Castells, Jaume M; Kurz, Andrea; Hallawa, Ahmed; Serpa Neto, Ary; Schultz, Marcus J; Gama de Abreu, Marcelo; Wittenstein, Jakob; IntelliLung Consortium, IntelliLung Consortium

---

VERSION 1 - REVIEW

**Reviewer**	**1**
**Name**	**Tosun, Betul**
**Affiliation**	**Hacettepe University, Faculty of Nursing**
**Date**	**04-Jun-2026**
**COI**	**None**

Thank you for submitting your manuscript entitled “Intelligent Lung Support in the Intensive Care Unit (IntelliLung): Study Protocol for an International Observational, Prospective, Multicentre Study”.

The topic is timely and highly relevant, and the protocol describes an ambitious international multicentre initiative evaluating an artificial intelligence–based decision support system for mechanical ventilation. The manuscript is generally well written and the study procedures are described in considerable detail. However, after editorial assessment, several important methodological issues require clarification before the manuscript can be considered for publication.

First, the manuscript repeatedly refers to the study as a prospective “validation” of the IntelliLung AI-DSS. However, the primary endpoint assesses agreement between AI-generated recommendations and ventilator settings selected by healthcare providers.

Agreement with clinician-selected settings alone may not be sufficient to establish clinical validation of the system. The authors should clarify the validation framework being applied and discuss the limitations of using concordance with current clinical practice as the primary measure of validation.

Second, the clinical interpretation of the primary endpoint remains unclear. While the protocol describes how agreement will be quantified, it does not define what level of agreement would be considered acceptable, clinically meaningful, or indicative of successful performance. Additional justification for the selected primary endpoint and its clinical relevance is required.

Third, the manuscript would benefit from a more detailed description of the AI system itself. Although the use of reinforcement learning is mentioned, limited information is provided regarding model development, training data characteristics, external validation procedures, handling of missing data, model updating, and safeguards against algorithmic bias. Given that the study focuses on an AI-driven decision support system, greater transparency regarding the underlying model is necessary.

Finally, considering the involvement of commercial partners and the declared competing interests, the manuscript should provide additional clarification regarding the role of the developers and sponsor in data management, data analysis, interpretation of results, and publication decisions.

In view of these concerns, I invite you to revise the manuscript and provide a detailed point-by-point response addressing the issues outlined above.

I look forward to receiving your revised submission.

VERSION 1 - AUTHOR RESPONSE

Response to Reviewer:

Thank you very much for your thoughtful review of our manuscript and for recognising the relevance and ambition of the IntelliLung study. We greatly appreciate the time and care you have taken in identifying these important methodological points, which have helped us substantially improve the clarity and rigour of the manuscript.

Below we provide a detailed, point-by-point response to each of your comments, with the corresponding revisions made in the manuscript. We hope these changes adequately address your concerns and look forward to your further feedback.

Reviewer comment 1: The manuscript repeatedly refers to the study as a prospective "validation" of the IntelliLung AI-DSS. However, the primary endpoint assesses agreement between AI-generated recommendations and ventilator settings selected by healthcare providers. Agreement with clinician-selected settings alone may not be sufficient to establish clinical validation of the system. The authors should clarify the validation framework being applied and discuss the limitations of using concordance with current clinical practice as the primary measure of validation.

Response: We thank the reviewer for this important and well-founded comment, which has helped us sharpen the conceptual framing of the study. We fully agree that agreement with

clinician-selected settings alone does not establish clinical validation, and that the term "validation" required clarification.
We would like to clarify that the validation framework applied in this trial is one of technical and operational validation. This includes demonstrating that an algorithm developed and tested on retrospective data can generate stable and clinically plausible recommendations when confronted with prospectively acquired, real-time clinical data, without manual pre-processing. This is a necessary prerequisite step that must precede any assessment of clinical efficacy or effect on patient outcomes, which is explicitly beyond the scope of the present observational design and is the subject of planned future interventional trials. We have revised the Discussion to state this distinction explicitly.
Regarding the choice of comparator, we would respectfully note that no true gold standard for mechanical ventilation settings exists. International guidelines including lung-protective and ARDSNet-derived strategies frequently lack precisely defined parameter thresholds and leave substantial latitude to clinician judgement. Clinician-selected settings, by contrast, represent a clearly defined, unambiguous, and clinically meaningful treatment strategy that was actually delivered to patients. They therefore constitute a pragmatic and interpretable reference for characterising the real-time behaviour of the AI-DSS, even though they are not, and are not treated as, a measure of ground-truth correctness.
Finally, we emphasise that the primary agreement endpoint is only one element of a deliberately broader evaluation. The secondary and exploratory analyses quantifying the magnitude and direction of deviations, benchmarking recommendations against guideline boundaries, identifying "missed opportunities", and exploring associations between agreement and patient-centred and clinical outcomes should provide a considerably more comprehensive assessment of the system than concordance alone. We have revised the Discussion (lines 634 to 643) to clarify the validation framework and to explicitly acknowledge the limitations of concordance with clinical practice as a measure of validation.

Reviewer comment 2: The clinical interpretation of the primary endpoint remains unclear. While the protocol describes how agreement will be quantified, it does not define what level of agreement would be considered acceptable, clinically meaningful, or indicative of successful performance. Additional justification for the selected primary endpoint and its clinical relevance is required.
Response: We thank the reviewer for this comment and welcome the opportunity to clarify the rationale for the primary endpoint.
The primary endpoint is, by design, estimative and exploratory rather than confirmatory. As stated in the Statistical Analysis section, no statistical hypotheses are tested in this observational trial. Accordingly, we have deliberately not pre-specified a threshold of agreement denoting "successful" performance. We would respectfully argue that defining such a threshold would not be scientifically justifiable: There is no empirical or guideline-based foundation for declaring a particular percentage of concordance between an AI-DSS and clinician practice to be clinically adequate.
This is reinforced by our argument (in response to Comment 1) that current clinical practice is not a subjective ground-truth and, and that complete concordance is therefore neither expected nor necessarily desirable.
The purpose of the primary endpoint is instead to precisely estimate the distribution of agreement for each of the four ventilator parameters, yielding the first quantitative characterisation of how closely a retrospectively trained AI-DSS might reproduce potential patterns of real-time expert decision-making across multiple ICUs. The required precision for this estimation drove the sample size calculation. The study is powered to estimate the distribution-free confidence interval for the median agreement of each parameter to a pre-

defined accuracy (e.g., expected precision of ±6.8% for FIO_2, ±2.4% for PEEP, and ±2.9% for Respiratory Rate (RR)), rather than to detect a difference against a hypothesised value. The clinical relevance of the primary endpoint is established not by the agreement value in isolation, but through the pre-planned exploratory analyses that relate the degree of AI-DSS-clinician agreement to patient-centred outcomes, most notably ventilator-free days at day 30 and mortality (in-hospital, day 30, day 180). This outcome-anchored approach allows us to assess whether, and in which direction, the system's behaviour is associated with clinically meaningful endpoints. We consider this hypothesis-generating design appropriate and necessary as a first step, the results of which will directly inform the design and power calculations of subsequent interventional trials assessing the effect of the AI-DSS on patient outcomes.
We have revised the discussion to make the estimative nature of the primary endpoint, the rationale for not defining an agreement threshold, and the outcome-anchored basis for its clinical relevance explicit.

Reviewer comment 3: The manuscript would benefit from a more detailed description of the AI system itself. Although the use of reinforcement learning is mentioned, limited information is provided regarding model development, training data characteristics, external validation procedures, handling of missing data, model updating, and safeguards against algorithmic bias...
Response: We thank the reviewer for this constructive comment and agree that greater transparency regarding the underlying model is warranted. We have substantially expanded the manuscript with a new "Medical Device Description" section addressing each of the points raised:
• Model development: We now specify that the recommendation algorithm is a factored conservative Q-learning (CQL) reinforcement-learning model and describe its development under IEC 62304:2016 and EU Regulation 2017/745. The source code of the model is available on GitHub (https://github.com/NIMI-research/INTELLILUNG_2)
• Training data characteristics: The training cohort, derived from the MIMIC-IV, eICU and HiRID datasets, is now characterised in detail in Table 2, alongside the state-vector definition (Table 3). Further methodological details and performance assessment results will be published in another publication currently prepared for submission to a Journal. As we do not want to jeopardize this publication, we will make the data and results available at OpARA (https://opara.zih.tu-dresden.de) after successful publication.
• External validation procedures: We have clarified that the model underwent retrospective internal validation, including medical evaluation of the action-frequency distribution on the held-out test set (Figure 2). At this stage, we would prefer not to report the results of the external validation in this protocol paper, as these are intended to be published as part of a dedicated development/validation manuscript and we would not want to pre-empt or jeopardise that publication. We have therefore described the existence and design of the external validation step in the Medical Device Description section, without presenting outcome data at this point. Should the reviewer consider this insufficient, we would be glad to discuss with the editors how best to accommodate further detail.
• Handling of missing data: We now report the proportion of affected timepoints and the imputation strategy applied (see Table 4).
• Model updating: We have added an explicit statement that the deployed model is locked for the duration of the trial, with no online learning or updates during data collection, ensuring reproducibility; any future revision would constitute a new device version requiring separate validation.
• Safeguards against algorithmic bias: We now describe the use of a large, multi-source,

multi-national training cohort to reduce single-centre dependence, external validation on an independent cohort, and the role of the trial's broad eligibility criteria in enabling prospective subgroup-specific performance assessment.
We would additionally like to note that a comprehensive description of model development and retrospective validation, including the full training procedure, hyperparameters, missing-data handling, and internal and external validation results, is beyond the scope of this protocol paper and is reported in a dedicated methods publication (in preparation), which we will cross-reference upon acceptance. The expanded device description provides the transparency necessary to interpret the present protocol, while directing readers to the forthcoming paper for full technical detail.

Reviewer comment 4: Finally, considering the involvement of commercial partners and the declared competing interests, the manuscript should provide additional clarification regarding the role of the developers and sponsor in data management, data analysis, interpretation of results, and publication decisions.
Response: We thank the reviewer for raising this point. We have added a statement clarifying the respective roles of all parties. The sponsor (Technische Universität Dresden) is an academic institution responsible for the overall design and conduct of the study. Data management, data analysis, interpretation of results, and all publication decisions rest exclusively with the academic investigators. The commercial partner BetterCare S.L. provided and maintains the data integration platform connecting hospital IT infrastructure to the secured platform, but had no role in study design, data analysis, interpretation, or the decision to publish. This is now stated explicitly in the Competing Interests section.
